# Diversification of *Rice Yellow Mottle Virus* and Related Viruses Spans the History of Agriculture from the Neolithic to the Present

**DOI:** 10.1371/journal.ppat.1000125

**Published:** 2008-08-15

**Authors:** Denis Fargette, Agnès Pinel-Galzi, Drissa Sérémé, Séverine Lacombe, Eugénie Hébrard, Oumar Traoré, Gnissa Konaté

**Affiliations:** 1 Institut de Recherche pour le Développement (IRD), UMR RPB, Montpellier, France; 2 Institut de l'Environnement et de Recherches Agricoles (INERA), Laboratoire de Biotechnologie et de Virologie Végétale, Kamboinsé, Ouagadougou, Burkina Faso; 3 Institut de Recherche pour le Développement (IRD), UMR GDP, Montpellier, France; The Pennsylvania State University, United States of America

## Abstract

The mechanisms of evolution of plant viruses are being unraveled, yet the timescale of their evolution remains an enigma. To address this critical issue, the divergence time of plant viruses at the intra- and inter-specific levels was assessed. The time of the most recent common ancestor (TMRCA) of *Rice yellow mottle virus* (RYMV; genus *Sobemovirus*) was calculated by a Bayesian coalescent analysis of the coat protein sequences of 253 isolates collected between 1966 and 2006 from all over Africa. It is inferred that RYMV diversified approximately 200 years ago in Africa, i.e., centuries after rice was domesticated or introduced, and decades before epidemics were reported. The divergence time of sobemoviruses and viruses of related genera was subsequently assessed using the age of RYMV under a relaxed molecular clock for calibration. The divergence time between sobemoviruses and related viruses was estimated to be approximately 9,000 years, that between sobemoviruses and poleroviruses approximately 5,000 years, and that among sobemoviruses approximately 3,000 years. The TMRCA of closely related pairs of sobemoviruses, poleroviruses, and luteoviruses was approximately 500 years, which is a measure of the time associated with plant virus speciation. It is concluded that the diversification of RYMV and related viruses has spanned the history of agriculture, from the Neolithic age to the present.

## Author Summary

The timescale of the evolution of plant viruses is an enigma, and even its order of magnitude is unknown. This critical issue is addressed here by calculating the age of plant viruses. An accurate estimate of the age of *Rice yellow mottle virus* (RYMV) was obtained by statistical analysis of a set of dated sequences. The age of RYMV provides a reliable calibration of related viruses, applying recently developed relaxed molecular clock models. It was found that RYMV diversified approximately 200 years ago, and that inter-specific diversification ranged from 500 years to 9,000 years. Altogether, plant virus diversification has spanned the history of agriculture from the Neolithic age to the present. This suggests that the Neolithic was a period of epidemiological transition for plant virus diseases, as already proposed for infectious human diseases. Intrinsically, it is for the same reason: increased contacts between hosts, pathogens, and vectors. This is consistent with the view that RNA viruses have a recent origin, and that humans have become the world's greatest evolutionary force.

## Introduction

The mechanisms of evolution of plant viruses are being progressively unraveled [1]–[3], yet the timescale of their evolution remains an enigma. Even the order of magnitude is unknown [4]. Several viruses showed few genetic changes between isolates separated in space and time, sometimes for centuries [5]–[8]. In contrast, recent evidence from statistical analyses of sequences of dated isolates of *Tomato yellow leaf curl virus* (genus *Geminivirus*) [9], *Rice yellow mottle virus* (genus *Sobemovirus*) (RYMV) [10] and *Zucchini yellow mosaic virus* (genus *Potyvirus*) [11] indicated rapid evolution, similar to that of most animal viruses. The paradox is addressed here by calculating the divergence time of plant viruses at the intra- and inter-specific levels using RYMV and related viruses.

Molecular-dating techniques provide insights into the history of lineages that have a poor or non-existent fossil record, such as viruses [12],[13]. These techniques were originally based on the assumption of a strict molecular clock reflecting steady accumulation of genetic changes over time. Recently, new methods enable the incorporation of variable rates into molecular dating [13]. Here, we applied a Bayesian Markov Chain Monte-Carlo method for performing relaxed phylogenies that is able to co-estimate phylogeny and divergence times under uncorrelated relaxed-clock models [14].

RYMV causes an emergent disease that was first observed in 1966 in Kenya. Since then, it has been reported in nearly all rice-growing countries of sub-Saharan Africa. RYMV is transmitted by coleopterous insects and is also disseminated abiotically. It has a narrow host range limited to wild and cultivated rices and a few related grasses [15]. There is no evidence of recombination between RYMV isolates [16],[17]. The rate of evolution of RYMV was recently evaluated using the coat protein (CP) sequences of 253 isolates collected between 1966 and 2006 from all over Africa [10]. The same group of sequences is analyzed here to assess the time of their most recent common ancestor (TMRCA), which is a measure of the divergence time of RYMV. The TMRCA was calculated by a Bayesian coalescent analysis of the sequences using several molecular clock and population genetic models [14].

Sobemoviruses infect both monocotyledonous and dicotyledonous plants, but the host range of each virus species is narrow and confined to a few plant species of the *Poaceae* or *Fabaceae*. Sobemoviruses are transmitted by beetle vectors, seeds and direct contact [18]. They share a common genomic organization, as found after re-sequencing some of the virus species [19],[20]. Ten sobemovirus species have been fully sequenced, nine of them are currently registered by ICTV [18] and a tentative one, Imperata yellow mottle virus (IYMV), was recently isolated from *Imperata cylindrica* in Africa [56]. Their genomes contain four open reading frames (ORFs). ORF1, located at the 5′ end of the genome, encodes a protein involved in virus movement and gene silencing suppression. ORF2 comprises two overlapping ORFs. ORF2a encodes a serine protease and a viral-genome-linked protein. ORF2b is translated through a -1 ribosomal frameshift mechanism through a fusion protein. It encodes the RNA-dependent RNA polymerase (RdRp). The coat protein gene (ORF4) is expressed by a sub-genomic RNA at the 3′ end of the genome. No evidence of recombination between sobemoviruses has been found either in phylogenetic [21],[56] or experimental studies [22].

The genus *Sobemovirus* is not assigned to a family. However, the RdRp of the sobemoviruses is phylogenetically related to that of the poleroviruses and enamoviruses (family *Luteoviridae*) [23], and to Poinsettia latent virus (PnLV), a putative polerovirus-sobemovirus hybrid [24]. Sobemoviruses, luteoviruses (family *Luteoviridae*) and dianthoviruses (family *Tombusviridae*) are more distantly related. The CPs of sobemoviruses are related to those of the necroviruses (family *Tombusviridae*), and the CPs of the poleroviruses to those of the luteoviruses [18]. Recombination events between ancestors of these genera are the likely causes of the present situation [25],[26]. Altogether, this led to the proposal of a “supergroup” to include these related genera [25]. The RdRp of the sobemoviruses also shows similarities with that of *Mushroom bacilliform virus* (MBV) (genus *Barnavirus*, family *Barnaviridae*) which infects mushrooms [27].

The divergence time of sobemoviruses was assessed from the full-length sequences using the age of RYMV under relaxed molecular clock models for calibration. The divergence time of the sobemoviruses with members of related genera was inferred from RdRp sequences with the same methodology. The time associated with plant virus speciation was assessed by calculating the TMRCA of closely related pairs of sobemoviruses, poleroviruses and luteoviruses. Collectively, these studies provide estimates of the diversification time of a plant virus species, the time associated with plant virus speciation, and the TMRCA of plant viruses of the same genus and of different genera. The intra- and inter-specific plant virus diversification was found to span the history of agriculture from the Neolithic age to the present.

## Results

### TMRCA of RYMV

The estimates of the TMRCA of RYMV inferred from the 253 dated CP sequences were dependent on both molecular clock and demographic models. Models enforcing relaxed molecular clocks performed better than the strict clock model, whatever the population genetic model selected (Table 1). The average substitution rates ranged from 5.1×10^−4^ to 12.3×10^−4^ nucleotides (nt)/site/year among the models (data not shown). The highest marginal likelihood was obtained with the model implementing the relaxed uncorrelated exponential molecular clock and the exponential growth model. The Bayes Factor (BF) gave strong support to this model when compared to other clock and population models. Under this model, the average TMRCA of RYMV was 195 years and the substitution rate was 11.7×10^−4^ nt/site/year. The median was 182 years. The highest density probability (HPD) interval ranged from 107 years to 308 years, with an approximate lognormal distribution of the estimates. Subsequently, a lognormal distribution with a lognormal mean of 5.2 and a standard deviation of 0.3 was applied as the prior distribution of TMRCA of RYMV for the upward calibration of nodes of sobemoviruses and related viruses.

**Table 1:** Estimates of the time of the most recent common ancestor (TMRCA) of *Rice yellow mottle virus* by Bayesian coalescent methods under several molecular clock and population genetic models implemented in BEAST

	Population Genetic Models											
	Constant Size			Exponential			Expansion			Bayesian Skyline		
Molecular Clock Model	Lower	Mean	Upper	Lower	Mean	Upper	Lower	Mean	Upper	Lower	Mean	Upper
Strict												
TMRCA (years)^a^	281	382	512	257	373	520	263	381	496	257	388	535
Marginal likelihood^b^		−8791			−8788			−8791			−8784	
Relaxed (lognormal)												
TMRCA (years)	182	342	511	166	280	408	183	312	463	167	306	483
Marginal likelihood		−8714			−8722			−8719			−8720	
Relaxed (exponential)												
TMRCA (years)	129	287	537	107	195	308	119	245	422	88	236	432
Marginal likelihood		−8704			−8697			−8709			−8702	

a 95% lower, mean, and 95% upper values of the highest posterior density (HPD) interval that contains 95% of the marginal posterior distribution.

b Marginal likelihoods, in log_e_ units, were calculated via importance sampling using the harmonic mean of the sampled likelihoods (with the posterior as the importance distribution).

### TMRCA of sobemoviruses and related viruses

**Figure 1:**
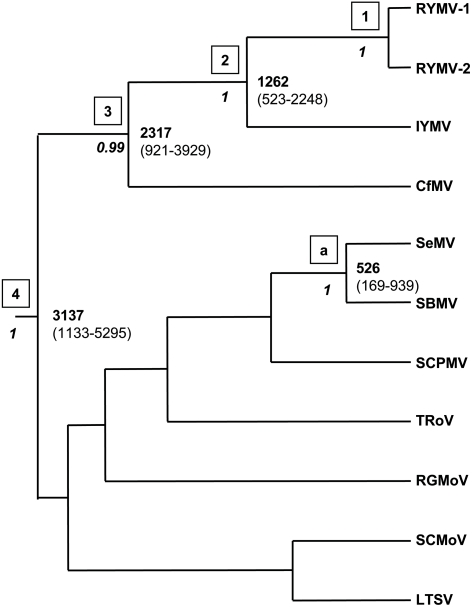
Divergence times of RYMV and sobemoviruses. The tree was reconstructed from the full sequences by Bayesian inference under an uncorrelated lognormal relaxed molecular clock model. The age of RYMV was used for calibration (node 1). Nodes 2–4 are associated with more internal nodes. External node “a” gathers SeMV and SBMV, the two most closely related sobemoviruses. The posterior probabilities are below the nodes (italics). The divergence times (in years) are positioned at the nodes, and the 95% HPD intervals are indicated in brackets. The species names and the sequence accession numbers are given in Table 1.

The two most divergent RYMV isolates, Ma10 and Tz202, were collected 5,000 km apart in Mali and Tanzania, respectively, and differed by 10.5% in the full genome. They were subsequently referred to as isolates RYMV-1 and RYMV-2. The distribution of the estimates of the TMRCA of RYMV calculated from the dated CP sequences was taken as the prior of their divergence time (node 1 in all figures). The full sequences of these two RYMV isolates and of nine other sobemoviruses were considered (Table 2). A total of 4,798 characters was analyzed, 3,432 of them (72%) being parsimony-informative. The lognormal clock model performed better than the strict model (marginal likelihoods in log_e_ units were −50237 and −50260, respectively), whereas the exponential model failed to converge. The deviation from the hypothesis of a strict clock was limited (coefficient of variation = 0.23). The TMRCA and the substitution rates of the sobemoviruses under the lognormal and the strict clock models were close: 3,137 vs. 3,326 years, and 4.0×10^−4^ vs. 3.7×10^−4^ nt/site/year, respectively. The Yule speciation process and the constant population size coalescent model as tree priors yielded similar estimates. Among sobemoviruses, RYMV is most closely related to IYMV (node 2). The TMRCA of RYMV and IYMV was 1262 years (523–2248) (Figure 1). *Cocksfoot mottle virus* (CfMV, genus *Sobemovirus*) also infects monocotyledonous plants but without overlap in host or geographical range. CfMV is the species the most closely related to RYMV and IYMV (node 3). The TMRCA of CfMV, IYMV and RYMV was 2,317 years (921–3,929). The root height of all sobemoviruses (node 4) was 3,137 years (1,133–5,295).

**Table 2:** Name, abbreviation, taxonomy and accession number of the virus species analyzed.

Name	Abbreviation	Genus	Family	Accession Number
*Cocksfoot mottle virus*	CfMV	*Sobemovirus*	Not assigned	DQ680848
*Imperata yellow mottle virus*	IYMV	*Sobemovirus*	Not assigned	AM990928
*Lucerne transient streak virus*	LTSV	*Sobemovirus*	Not assigned	NC001696
*Rice yellow mottle virus*	RYMV-1	*Sobemovirus*	Not assigned	AJ608208
	RYMV-2			AM883057
*Ryegrass mottle virus*	RGMoV	*Sobemovirus*	Not assigned	EF091714
*Sesbania mosaic virus*	SeMV	*Sobemovirus*	Not assigned	NC002568
*Southern bean mosaic virus*	SBMV	*Sobemovirus*	Not assigned	AFO55887
*Southern cowpea mosaic virus*	SCPMV	*Sobemovirus*	Not assigned	NC001625
*Subterranean clover mottle virus*	SCMoV	*Sobemovirus*	Not assigned	AY376451
				AY376452
*Turnip rosette virus*	TRoV	*Sobemovirus*	Not assigned	NC004553
*Beet chlorosis virus*	BChV	*Polerovirus*	*Luteoviridae*	NC002766
*Beet mild yellowing virus*	BMYV	*Polerovirus*	*Luteoviridae*	NC003491
*Cereal yellow dwarf virus-RPS*	CYDV-RPS	*Polerovirus*	*Luteoviridae*	NC002198
*Cereal yellow dwarf virus-RPV*	CYDV-RPV	*Polerovirus*	*Luteoviridae*	EF521848
*Potato leafroll virus*	PLRV	*Polerovirus*	*Luteoviridae*	AF453394
*Barley yellow dwarf virus-MAV*	BYDV-MAV	*Luteovirus*	*Luteoviridae*	D11028
*Barley yellow dwarf virus-PAS*	BYDV-PAS	*Luteovirus*	*Luteoviridae*	NC 002160
*Red clover necrotic mosaic virus*	RCNMV	*Dianthovirus*	*Tombusviridae*	JO4357
*Mushroom bacilliform virus*	MBV	*Barnavirus*	*Barnaviridae*	NC 001633

The divergence time of sobemoviruses and related viruses was assessed from the RdRp sequences (Table 2). Again, the distribution of the estimates of the TMRCA of RYMV calculated from the dated CP sequences was taken as the prior of the divergence time of RYMV-1 and RYMV-2 (node 1). A total of 2,199 characters were analyzed, 1,607 being parsimony-informative (73%). The model enforcing the lognormal clock model performed better than the strict model (marginal likelihoods in log_e_ units were −29663 and −29677, respectively), whereas the exponential model failed to converge. Again, the deviation from the hypothesis of strict clock was limited (coefficient of variation = 0.28) and the estimates were close. For instance, the basal root height under the lognormal and the strict clocks were 8,772 vs. 10,440 years, and the substitution rates were 3.2**×**10^−4^ and 2.8**×**10^−4^ nt/site/year, respectively. However, the HPD interval was wider with the lognormal model (2,929–15,671 years) than with the strict model (4,971–18,060 years), i.e., a 1∶5.4 ratio for the lognormal model vs. a 1∶3.6 ratio for the strict clock model.

**Figure 2:**
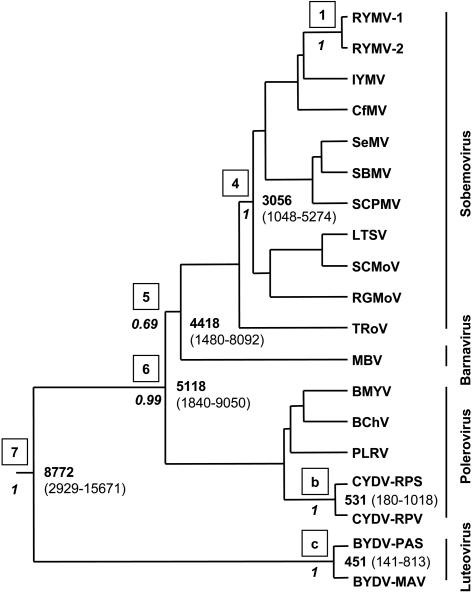
Divergence times of sobemoviruses and related viruses. The tree was reconstructed from the RdRp sequences by Bayesian inference under an uncorrelated lognormal relaxed molecular clock model. The age of RYMV was used for calibration (node 1). Nodes 4–7 are associated with more internal nodes. External node “b” gathers CYDV-RPS and CYDV-RPV, the two most closely related poleroviruses. External node “c” gathers BYDV-PAS and BYDV-MAV, the two most closely related luteoviruses. The posterior probabilities are below the nodes (italics). The divergence times (in years) are positioned at the nodes, and the 95% HPD intervals are indicated in brackets. The species genus is indicated alongside the vertical line. The species names and the sequence accession numbers are given in Table 1.

**Figure 3:**
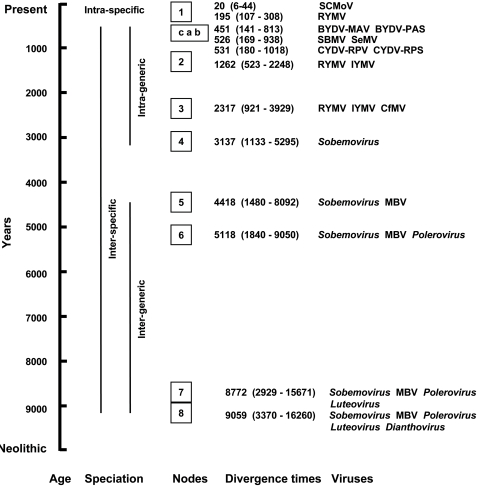
Divergence times of RYMV, sobemoviruses, and related viruses. The divergence times and the 95% HPD intervals are in brackets and framed. Nodes 1 to 8 encompass plant virus diversification at the intra-specific, intra- and inter-generic levels, as indicated by the vertical lines. Nodes “a,” “b,” and “c” gather closely related pairs of viruses. The time axis spreads from the beginning of the Neolithic period to the present.

The age of sobemoviruses (node 4) calculated on the full genome and on the RdRp sequences were similar (3,137 and 3,056 years, respectively) despite the difference in number of parsimony-informative characters considered (3,432 vs. 1,607 characters). The age of sobemoviruses calculated on the CP sequences was similar (2,884 years) although the dN/dS ratios of the RdRp and of the CP genes were 0.18 and 0.39, respectively, reflecting the differences in functional constraints operating on the two genes. The TMRCA of the sobemoviruses and MBV (node 5) was 4,418 years (1,480–8,092) (Figure 2). The divergence time of sobemoviruses, poleroviruses, and MBV (node 6) was 5,118 years (1,840–9,050). The root height of sobemoviruses, MBV, poleroviruses, and luteoviruses (node 7) was 8,772 years (2,929–15,671). The TMRCA of these viruses and *Red clover necrotic mosaic virus* (RCNMV) (genus *Dianthovirus*) was 9,059 (3,370–16,260) (node 8), a value not substantially different from node 7 (Figure 3).

### TMRCA of closely related virus species

Several isolates of *Subterranean clover mottle virus* (SCMoV, genus *Sobemovirus*), which caused a disease restricted to southwest Australia, were fully sequenced [28]. The highest divergence between two isolates collected in 1991 and 1996, respectively, was 1.2%. Accordingly, the divergence time of SCMoV was estimated to be 20 years with a HPD interval of 6–44 years, indicating a date of diversification between 1952 and 1990 (Figure 3). *Southern bean mosaic virus* (SBMV, genus *Sobemovirus*) and *Sesbania mosaic virus* (SeMV, genus *Sobemovirus*) differed by 31.6% in their complete genome and thus are the two most closely related sobemoviruses (Figure 1). Their divergence time (node “a”) was 526 years (169–938). *Cereal yellow dwarf virus* CYDV-RPV and CYDV-RPS, two closely related poleroviruses, differed by 22% in their RdRp sequences (Figure 2). Their TMRCA was 531 years (180–1,018) (node “b”). *Barley yellow dwarf virus* BYDV-MAV and BYDV-PAS, two closely related luteoviruses, differed by 21.1% in their RdRp. Their divergence time (node “c”) was 451 years (141–813). Altogether, the TMRCA of these closely related pairs of sobemoviruses, poleroviruses and luteoviruses ranged from approximately 450 to 550 years.

## Discussion

The 253 RYMV isolates collected in 16 countries represent the diversity of the species [10],[17]. Accordingly, the TMRCA of these 253 isolates provides a reliable estimate of the divergence time of RYMV. By contrast, the 10 sobemovirus species probably underestimate the number of sobemoviruses in cultivated and wild plants [29]. However, theoretical studies indicated that numerous samples are not necessary to date old coalescent events. It was calculated that the coalescence time of a sample of 10 taxa was 90% of the expected coalescent time of the entire population [30]. Consequently, although calculated on a limited number of species, the TMRCA of sobemoviruses and members of related genera provide reliable estimates of their divergence times.

Relaxed molecular clock models incorporate the rate variation among lineages in estimates of divergence time. Accordingly, any punctuated evolution, as might occur in species jump, should be accounted for in the relaxed clock models. Results from relaxed clocks should be evaluated in relation to those of strict clocks [31]. In our study, the lognormal relaxed clock model performed better at the inter-specific level than the strict clock model. However, the deviation from a strict clock model was limited. This explained why the TMRCA estimates under strict and relaxed clock models were close.

There was, however, a 1∶3 ratio between the lower and upper bounds of the HPD intervals of the TMRCA of RYMV (308 and 107 years, respectively). The variance of this estimate, further enlarged after relaxation of the molecular clock assumption, accounted for the large HPD intervals of divergence times at the inter-specific level. However, the HPD of RYMV divergence time is still substantially narrower than those of the other plant viruses studied with dated sequences [9],[11]. This is likely to be due to the larger number of isolates used and the wider range of dates encompassed with RYMV. This could also reflect the fact that the RYMV isolates were collected, sequenced and analyzed by the same group of scientists, subsequently reducing the uncertainties associated with the use of data sets from various and heterogeneous sources.

Assessing the divergence time of RYMV from dated sequences does not suffer from the limitations of alternative approaches. Measuring RYMV evolution rate from experimental studies or from old virus specimens was previously found to be inappropriate [10]. Applying epidemiological evidence is not adequate either. Symptoms of RYMV were first described in 1966, i.e., 40 years ago, a value inconsistent with the 107 to 308 years of the HPD interval for RYMV diversification. This means that RYMV diversified decades before the disease symptoms were reported. It also suggests that RYMV caused epidemics long before it was recognized as a disease. The first report of symptoms should better be considered as a lower bound of virus diversification, i.e., the minimum time since the virus diversified. Exceptions are viruses in localized and well-surveyed regions such as SCMoV in southwest Australia. From dated sequences, SCMoV diversification was estimated to occur between 1952 and 1990. This interval includes 1979, the year when the first symptoms were reported [32]. Biogeographical evidence to estimate divergence time can be misleading too. Madagascar was separated from mainland Africa approximately 100 millions years ago. The timescale of evolution of RYMV excludes the possibility that the divergence between isolates from Madagascar and from East Africa reflects vicariance events [33]. Altogether, the set of CP sequences of 253 dated isolates of RYMV currently provides the most reliable approach to date plant virus diversification.

The divergence time of RYMV was approximately 200±100 years, whereas symptoms were reported for the first time in 1966 in East Africa [34] and in 1975 in West Africa [35]. The African rice *Oryza glaberrima* was domesticated in West Africa approximately 3,000 years ago, whereas the Asiatic rice *O. sativa* was introduced in the 10^th^ and 16^th^ centuries in East and West Africa, respectively [36],[37]. Consequently, RYMV diversified centuries after rice was domesticated or introduced in Africa, and decades before epidemics were reported. The 19^th^ century was a period of extension of the rice culture in Africa [37]. This may have favored the spread of RYMV from is primary host to rice, followed by its dissemination throughout Africa.

The divergence time between sobemoviruses and related viruses was estimated to be approximately 9,000 years, that between sobemoviruses and poleroviruses approximately 5,000 years, and that among sobemoviruses approximately 3,000 years (Figure 3). The estimates of the age of sobemovirus diversification did not depend on the sequence length or on the gene considered. Even considering their HPD, these time-scales encompassed the Neolithic “agricultural revolution.” This period was the transition from nomadic hunting and gathering communities to agriculture and settlement. It occurred independently in several prehistoric human societies between 10,000 and 4,000 years before present (BP) [38],[39]. Ancient peoples completed the domestication of all major plant species upon which human survival depends ca. 4,500 years BP [40],[41].

One likely consequence of agricultural expansion is the dramatic increase of opportunities for encounters between wild and cultivated plant species, between cultivated plants at various stages of domestication, and between plants and potential insect vectors. These new encounters must have facilitated the emergence of plant viruses. This is still apparent nowadays when crop species are moved from their center of origin into new regions. They are exposed to infection by indigenous viruses to which they have not previously been adapted [4],[42],[43]. Further crowding of plants associated with agricultural development, especially monoculture, facilitated the build-up of vector populations and the disease spread, as is still apparent at the present time [43]. Similarly, the Neolithic age was critical for the emergence of infectious human diseases, a period referred to as the first epidemiologic transition [44]. This was attributed to the increased contacts between humans and wild fauna, and among humans themselves. Our results suggest that the Neolithic age was also a period of epidemiological transition for plant pathogens such as viruses, intrinsically for the same reason: increased contacts between hosts, pathogens and vectors. The hypothesis that the emergence of plant viruses is linked to the development of agriculture is consistent with the view that RNA viruses have a recent origin [12], and also that humans have become the world's greatest evolutionary force [45].

The divergence time of the RdRp of sobemoviruses and poleroviruses bounded the dates of the recombination events between the genera. They must have occurred after the diversification of the common ancestor of the RdRp of sobemoviruses and poleroviruses approximately 5,000 years ago, and before the diversification of each of the two genera approximately 3,000 years ago. These recombination events, which necessarily involved the co-existence of different genomes in the same plant, must have been favored by the increased opportunities of co-infections associated with agricultural expansion that started during the Neolithic age. Events occurring at this period also possibly led to virus diversification outside the plant kingdom, as suggested by the divergence time of the sobemovirus and MBV estimated to be approximately 4,500 years.

Much effort has been recently devoted to the numerical taxonomy of plant viruses to set thresholds in percentage of nucleotide divergence for demarcation criteria at the intra- and inter-specific levels [46]. In this study, nucleotide divergence illuminates the timescales associated with these demarcation criteria (Figure 3). The limited deviation from the strict clock model allowed the comparison of these timescales. The inter-generic divergence time between sobemoviruses, poleroviruses and luteoviruses exceeded approximately 3,000 years. The inter-specific divergence of sobemoviruses ranged from approximately 500 to 3,000 years. Consistent divergence times of approximately 500 years were obtained between closely related pairs of sobemoviruses, luteoviruses and poleroviruses, which were first considered as strains and later ranked as different species. This provides an estimate of the time associated with speciation of plant viruses. The intra-specific divergence time of RYMV was approximately 200 years, which is 2 to 3 times less than the speciation time of plant viruses. Overall, this range of values revealed that plant diversification at the intra- and inter-specific levels occurred within the Holocene, and has spanned the entire history of agriculture, from the Neolithic age to the present.

## Materials and Methods

### Sequence analyses

The CP genes (720 nucleotides) of 253 isolates from 16 countries in Africa collected over a 40-year period, and the complete genome of two isolates of RYMV were previously sequenced [10],[17]. The complete sequences of the sobemoviruses, the sequences of the RdRp of the poleroviruses, luteoviruses, PnLV, and MBV were downloaded from GenBank (Table 1). The sequences were aligned using CLUSTAL W with default parameters [47]. The parameters of interest were estimated within a Bayesian coalescent framework by a Markov Chain Monte Carlo (MCMC) method using the Bayesian Evolutionary Analysis by Sampling Trees (BEAST) program (http://beast.bio.ed.ac.uk/) [48]. The Bayesian MCMC method estimates a parameter as the mean of its posterior distribution while simultaneously incorporating uncertainty in the underlying genealogy or phylogeny and other parameters.

The length and number of MCMC chains were chosen so that the effective sample size for the root height parameter and other parameters was >200, indicating that the parameter space was sufficiently explored. The convergence of the parameters to a stationary distribution was assessed with TRACER [49], and the statistical uncertainties were summarized in the 95% HPD intervals. Comparison of models was performed by calculating the Bayes Factor (BF), which is the ratio of the marginal likelihood of each model [50]. A value of log_e_(BF) >2.3 was taken as evidence of a strong support for the model with the highest marginal likelihood. The coefficient of variation of the evolution rates calculated under the uncorrelated lognormal relaxed clock model was used to assess the degree of deviation from the strict molecular clock model.

### TMRCA of RYMV

In earlier studies, the evolution rate was the target parameter [10], whereas here the TMRCA or the root height was the parameter of interest. It was taken as a measure of the divergence time of RYMV. The root height was estimated by enforcing strict and relaxed (uncorrelated lognormal and uncorrelated exponential) molecular clocks as implemented in BEAST [48]. Four demographic models were applied as coalescent priors: constant population size, exponential growth, expansion growth, and a piece-wise Bayesian skyline plot [49]. Default values were used for the other priors. The uncertainty in the TMRCA of RYMV is summarized by the highest posterior density interval that contains 95% of the marginal posterior distribution.

### TMRCA of sobemoviruses and related viruses

The full sequences of 10 sobemoviruses were considered for the intra-generic analysis (Table 2). The RdRp sequences of related viruses were added for the inter-generic analysis. The total number of characters and the number of parsimony informative characters were calculated with PAUP [51]. The dN/dS ratios were calculated under the MG94 model [52] as implemented in Hyphy (http://www.hyphy.org/) [53]. The poleroviruses listed by ICTV [23], *Pea enation mosaic virus* (genus *Enamovirus*) and PnLV were screened for recombination signals. Putative recombinant genomes were searched using the RDP3 package (http://darwin.uvigo.es/rdp/rdp.html). It implements six recombinant detection programs: RDP, GENECONV, MaxChi, Chimera, Bootscan and Siscan [54]. The default detection thresholds were applied. Five poleroviruses showing no signals of recombination were subsequently selected: *Beet chlorosis virus* (BchV), *Beet mild yellowing virus* (BMYV), *Potato leaf roll virus* (PLRV), CYDV-RPS and CYDV-RPV (Table 2). Similarly, the RdRP sequences of two luteoviruses were chosen: BYDV-PAS and BYDV-MAV.

The best-fitting nucleotide substitution model was evaluated by hierarchical likelihood ratio testing [55], as implemented in HyPhy [53]. The best-fitting model was the HKY model with gamma rate heterogeneity. The dates of isolation of the virus species were considered as contemporaneous as they differed by a few years only, whereas our study dealt with inter-specific divergence times ranging from hundreds to thousands of years. The maximum clade credibility tree was reconstructed by Bayesian inference under the relaxed molecular clock models as implemented in BEAST. A Yule speciation process was selected as a tree prior. The distribution of the estimates of the TMRCA of RYMV was subsequently used as the prior of the RYMV node for upward calibration of the nodes of the trees. The HPD intervals of the TMRCA of sobemoviruses and related viruses subsequently summarized both the uncertainties of the phylogenetic signal and of the prior (the RYMV age). A uniform distribution with bounds of 5×10^−5^ and 5×10^−3^ nt/site/year was applied as the prior of the uncorrelated lognormal relaxed clock mean. A similar prior was applied for the Yule speciation process birth rate. A uniform distribution with bounds of 0.2 and 5 was applied as the prior of the gamma shape parameter. A Jeffrey prior with initial value of 1 was applied for the HYK transition-transversion parameter.
